# Serum Amyloid A1 Induces Classically Activated Macrophages: A Role for Enhanced Fibril Formation

**DOI:** 10.3389/fimmu.2021.691155

**Published:** 2021-06-30

**Authors:** Ann-Kathrin Gaiser, Shanna Bauer, Stephanie Ruez, Karlheinz Holzmann, Marcus Fändrich, Tatiana Syrovets, Thomas Simmet

**Affiliations:** ^1^ Institute of Pharmacology of Natural Products and Clinical Pharmacology, Ulm University, Ulm, Germany; ^2^ Institute of Protein Biochemistry, Ulm University, Ulm, Germany; ^3^ Genomics Core Facility, Medical Faculty, Ulm University, Ulm, Germany

**Keywords:** amyloidosis, macrophage polarization, M1 subset, serum amyloid A, inflammation, innate immunity

## Abstract

AA amyloidosis belongs to the group of amyloid diseases which can follow chronic inflammatory conditions of various origin. The disease is characterized by the deposition of insoluble amyloid fibrils formed by serum amyloid A1 (SAA1) leading eventually to organ failure. Macrophages are intimately involved in the fibrillogenesis as well as in the clearance of amyloid fibrils. *In vivo*, macrophages may occur as classically (M1) or alternatively activated (M2) macrophages. We investigate here how SAA1 might affect the macrophage phenotype and function. Gene microarray analysis revealed upregulation of 64 M1-associated genes by SAA1. M1-like polarization was further confirmed by the expression of the M1-marker MARCO, activation of the NF-κB transcription factor, and secretion of the M1-cytokines TNF-α, IL-6, and MCP-1. Additionally, we demonstrate here that M1-polarized macrophages exhibit enhanced fibrillogenic activity towards SAA1. Based on our data, we propose reconsideration of the currently used cellular amyloidosis models towards an *in vitro* model employing M1-polarized macrophages. Furthermore, the data suggest macrophage repolarization as potential intervention strategy in AA amyloidosis.

## Introduction

Systemic AA amyloidosis is a protein misfolding disease that is characterized by the deposition of amyloid fibrils in multiple organs. Amyloid fibrils consist of polypeptide aggregates with a cross-β structure that are deposited mainly in spleen, liver, and kidneys (1). Final consequences of amyloid deposition can be as severe as end-stage renal diseases with sometimes fatal outcomes (1). In addition, AA amyloidosis is transmissible. Mice with increased plasma SAA levels exhibited accelerated AA amyloidosis by seeding with AA fibrils from a number of other species indicating cross-species transmission, but also interspecies similarities (1).

There are three genes in humans that encode serum amyloid A (SAA) proteins (2), but only two of them, SAA1 and SAA2 are acute phase proteins meaning their expression is highly induced by inflammatory cytokines during acute phase response (1). Furthermore, only SAA1 protein that contains a more hydrophobic N-terminal region compared to other SAA isoforms is found in fibrils in systemic AA amyloidosis (2). Mouse SAA proteins are highly homologous to the corresponding human counterparts. Thus, mouse SAA1 is highly amylogenic and is often used to study AA amyloidosis *in vivo* (2–4). The major difference between human SAA proteins concerns SAA3. Thus, mouse *Saa3* product is produced by extrahepatic tissues including macrophages, whereas the human *Saa3* is a pseudogene (2). Different to SAA1, mouse SAA3 is nonamyloidogenic (2).

Physiologically, SAA1 protein with a normal serum concentration of 0.001 mg/ml may exceed more than 1 mg/ml in the acute phase response during a systemic reaction to tissue injury, infection, or trauma (5). Such elevated SAA1 plasma concentrations set the stage for the development of AA amyloidosis, especially if they remain sustained over a prolonged period of time. Hence, AA amyloidosis is often associated with chronic inflammatory conditions, such as rheumatoid arthritis (1). The mechanism of amyloid fibril formation is still not completely understood but macrophages critically contribute to amyloid formation *in vivo* (6). *In vitro* analysis of both, mouse and human macrophages, supported their role in amyloidogenesis (1). Yet, macrophages are also involved in the clearance of amyloid fibrils (7). Whether macrophages promote or prevent amyloid formation depends on the SAA1 load and its binding to HDL, as well as several other factors, such as the macrophage general metabolic activity (3, 7, 8). However, the complex biology of macrophages has largely been neglected when studying the formation of amyloid deposits.

Macrophages represent a very heterogeneous cell population with various subsets fulfilling diverse functions. On the one hand, proinflammatory classically activated M1 macrophages produce chemokines and cytokines for the recruitment and activation of immune effector cells as well as for the polarization of T helper cells. On the other hand, upon resolution of the infection, the anti-inflammatory alternatively activated M2 subset regulates the immune response and promotes wound healing. In addition to these main categories, the M2 macrophage subset comprises a panel of different cell populations designated M2a, M2b, M2c, and M2d. *In vivo*, macrophages might occur as intermediates of these extremes and macrophage polarization towards either one of those subsets is not a definite step but a plastic and reversible process (9, 10).

Macrophages are not only crucially involved in the biogenesis of amyloid fibrils but might also be affected by SAA1. Few studies have addressed the effect of SAA1 on macrophage polarization, all describing a shift towards the M2 (11) or, more specifically, to the M2b subset characterized by the secretion of proinflammatory cytokines combined with anti-inflammatory IL-10 production and a lack of IL-12 formation (12). However, in most studies mouse macrophages or monocytic leukemia cell lines were exposed to hybrid hSAA1 or hSAA2 molecules not found in nature or to human recombinant SAA1 containing an N-terminal methionine and two amino acid substitutions in its sequence. Such preparations have been shown to induce cellular effects different to those induced by native human SAA1 (13). Moreover, in studies reporting a M2-polarizing effect of SAA1, rather low SAA concentrations of about 0.006 mg/ml were used (11, 12), concentrations found under physiologic, non-inflammatory conditions (5).

In the present study we have analyzed the effects of mouse recombinant SAA1 (previous nomenclature SAA2) devoid of LPS and exhibiting the correct primary structure (3) at a concentration commonly observed during an acute phase on primary peritoneal mouse macrophages to optimally reflect the physiology of amyloidogenesis.

## Materials and Methods

### Reagents and Equipment

LPS from the *E. coli* strain O55:B5 and polymyxin B were obtained from Sigma-Aldrich (Steinheim, Germany), staurosporine from Alexis Biochemicals (San Diego, CA). Premium grade mouse IFN−*γ*, and mouse IL-4 were from Miltenyi Biotec (Bergisch Gladbach, Germany).

### Cell Culture

Experiments were conducted with murine peritoneal macrophages, the murine macrophage cell line J774A.1 (ACC 170; DSMZ, German Collection of Microorganisms and Cell Cultures, Braunschweig, Germany), or the murine NF-κB-SEAP & IRF-Luc J774-Dual^™^ cells (j774d-nfis; Invivogen, San Diego, CA). All animal work was conducted in accordance with the institutional and European guidelines and approved by the Animal Welfare and Ethical Review Board. Resting peritoneal cells were isolated from the abdominal cavity of euthanized NMRI mice (Charles River Laboratories, Bad Könighofen, Germany) of 8-10 weeks (14). Peritoneal cells were collected with ice-cold PBS, transferred to 6-well plates (Corning, Vineland, NJ) at 5 × 10^6^ cells/well in DMEM/F12 GlutaMAX, 10% heat-inactivated FCS, 10 mM L-glutamine, and penicillin/streptomycin, and were allowed to adhere for 4 h at 37°C. Non-adherent cells were removed by washing with pre-warmed PBS. Expression of macrophage markers was analyzed in all peritoneal lavage cells and in macrophages purified by adhesion. As required, purified macrophages were scraped and seeded at 1 × 10^5^ cells/cm^2^. On day 2 post-isolation, purified macrophages were either left untreated (M0), treated with SAA1, or polarized with LPS O55:B5 (100 ng/ml) and IFN-*γ* (20 ng/ml) to the M1-like phenotype or with IL-4 (20 ng/ml) to the M2-like phenotype for additional 24 h (15–17). The J774A.1 macrophage cell line was cultured in DMEM containing 4 mM L-glutamine, 10% heat-inactivated FCS, 1 mM pyruvate, and penicillin/streptomycin and used at a density of 10^4^ cells/cm^2^. J774-Dual™ cells were cultured in DMEM containing 2 mM L-glutamine, 10% heat-inactivated FCS, 1 mM pyruvate, 4.5 g/l glucose, penicillin/streptomycin, and the antibiotics blasticidin and zeozin for selection.

### Preparation of SAA1

Murine full-length SAA1.1 protein was recombinantly expressed in *Escherichia coli* RV308 cells (3). Residual trifluoroacetate from the purification was removed by filtration through a 3-kDa membrane filter. Recombinantly expressed SAA1 is soluble and lacks the amyloid structure as shown by weak interaction with amyloid binding dyes, such as thioflavin T or Congo red (3). The SAA1 batches used did not contain any relevant levels of endotoxin (< 0.2 EU/mg SAA1 protein) as assessed by EndoLISA^®^ (Hyglos, Bernried, Germany). Alexa Fluor^®^ 488-labeled SAA1 (SAA1-AF488) was prepared with the Alexa Fluor 488 Microscale Protein Labeling Kit^®^ using 3.8 µl of the Alexa Fluor^®^ 488 TFP ester solution to label 100 µg SAA1 protein according to the manufacturer’s instructions.

### Cellular Metabolic Activity

Cellular metabolic activity as a measure for cellular viability, was analyzed by the RealTime-Glo™ MT Cell Viability Assay (Promega Corporation, Walldorf, Germany). Metabolically active cells reduce the prosubstrate to a NanoLuc^®^ luciferase substrate allowing for rapid detection of cell viability. Luminescence measurements were performed using the Tecan plate reader Infinite M1000 Pro (Tecan, Männedorf, Switzerland).

### Microarray‐Based Gene Expression Analysis

Resting peritoneal macrophages (day 2 post-isolation) were treated with 50 µM SAA1 or the corresponding amount of Ampuwa (LPS-free H_2_O) for 6 h or 24 h (n = 3 per group). Cells were rinsed with ice-cold PBS and harvested by scraping. Cells were spun down by centrifugation, supernatant was discarded, and the cell pellets were frozen in liquid nitrogen and stored at -80°C until RNA isolation was performed. RNA was isolated and the RNA integrity number (RIN) determined using an Agilent Bioanalyzer (Agilent Technologies, Santa Clara, CA). Samples with a RIN of ≥ 9.1 were used. Following RNA (200 ng) reverse transcription, amplification, and labeling, ssDNA was hybridized to Affymetrix^®^ Mouse Gene 2.0 ST Arrays (Affymetrix, Santa Clara, CA). Microarray analyses were performed using 5.5 μg ssDNA per hybridization in a GeneChip^®^ Fluidics Station 450 and scanned using a GeneChip^®^ scanner 3000 (both Affymetrix). Images were analyzed using Affymetrix Expression Console^®^ Software and BRB‐Array Tools (http://linus.nci.nih.gov/BRB-ArrayTools.html). Raw feature data were normalized and log_2_ intensity expression summary values for each probe set were calculated using the Robust Multi-array Average (RMA) method. Filtering: Genes showing minimal variation across the set of arrays were excluded from the analysis. Genes whose expression differed by at least 1.5 fold from the median in at least 20% of the arrays were retained. Class comparison: Genes were identified as differentially expressed among the two groups using a two sided *t*-test. Genes were considered statistically significant when their *p*-value was less than 0.05 [Benjamini–Hochberg false discovery rate, FDR < 0.1 (18)] and displayed a fold change between the two groups of at least 2-fold. Complete microarray data are available at Gene Expression Omnibus (GEO accession number: GSE155278).

### Characterization of Surface Markers by Flow Cytometry

Freshly isolated peritoneal cells or purified peritoneal macrophages and J774A.1 cells treated as described above, were harvested by scraping and stained with anti-F4/80 (Miltenyi Biotec, Bergisch Gladbach, Germany), anti-CD11b (Bio-Rad Laboratories, Feldkirchen, Germany), anti-MARCO (R&D Systems, Wiesbaden-Nordenstadt, Germany), anti-CD38 (BD Biosciences, San Jose, CA), anti-CD206 (BD Biosciences), or respective isotype controls for 20 min at 4°C. Flow cytometry was conducted on a FACSVerse instrument (BD Biosciences) and analysis was performed using FlowJo software (Ashland, OR).

### Microscopy

Bright field images of peritoneal and J774A.1 macrophages were taken in the DIC channel at the indicated time points after polarization using an Eclipse Ti-E fluorescence microscope (Nikon, Tokyo, Japan). For fluorescence microscopy, peritoneal macrophages were plated on Falcon 8-well culture slides (Corning) at a density of 10^5^ cells/cm^2^. Cell staining was performed on day 2 post-isolation using anti-F4/80 (Miltenyi Biotec), anti-CD11b (Bio-Rad Laboratories), or respective isotype controls for 15 min at 4°C. Samples were fixed with paraformaldehyde and mounted in Vectashield^®^ mounting medium with DAPI (Vector Laboratories, Burlingame, CA). Images were acquired in the DAPI, FITC, and PE channels using an Eclipse Ti-E fluorescence microscope (Nikon).

### Cellular Uptake of SAA1

The uptake of SAA1 by peritoneal macrophages was analyzed by flow cytometry and fluorescence microscopy. Cells were treated on day 2 post-isolation with 49 µM SAA1 and 1 µM SAA1-AF488 for up to 24 h. For flow cytometric analysis, cells were treated at the respective time points with Trypsin/EDTA for 5 min at 37°C to remove extracellularly bound protein. Intracellular SAA1 was analyzed flow cytometrically in the FITC channel. For fluorescence microscopy, cells were seeded in 8 well slides with 180 µm-thin hydrophilic µ-bottom (Ibidi, Gräfelfing, Deutschland). Images were taken in the FITC and DIC channel at the indicated time points.

### Quantification of Cytokines in Cell Culture Supernatants

Secretion of TNF-α, IL-6, MCP-1, IFN-*γ*, IL-12p70, and IL-10 cytokines was analyzed with the mouse inflammation cytometric bead array (CBA, BD Biosciences) by flow cytometry according to the manufacturer’s protocol. Cytokine concentrations were calculated using the FCAP Array Software (BD Biosciences). In addition, to validate the absence of IL-10 secretion, cell culture supernatants were analyzed by ELISA using the mouse IL-10 DuoSet ELISA (R&D Systems Inc.).

### Determination of NO Levels

The levels of reactive nitrogen species in the cell culture supernatant of stimulated cells was assessed using the Nitrate/Nitrite Fluorometric Assay Kit (Cayman Chemical, Ann Arbor, MI).

### Analysis of NF-κB and IRF Activation

Murine NF-κB-SEAP/IRF-Luc J774-Dual™ cells (InvivoGen, San Diego, CA) were seeded in triplicates at 5,600 cells/well in a 96-well plate and either treated with 50 µM SAA1, maintained as M0 macrophages, or polarized towards M1- or M2-like subsets by treatment with 100 ng/ml LPS O55:B5 plus 20 µg/ml IFN-*γ* or 20 µg/ml IL-4, respectively. After 24 h, activation of NF-κB and IRF were assessed by the addition of cell culture supernatant to the QUANTI-Blue detection reagent or luciferin substrate, respectively. The color reaction was allowed to proceed for 1 h before absorbance was measured at 620 nm.

### Congo Red and Thioflavin T Staining

Peritoneal macrophages were plated on glass cover slips (Thermo Fisher Scientific, Waltham, MA) at a density of 10^5^ cells/cm^2^. On day 2 post-isolation, SAA1 was added in fresh medium to a final concentration of 25 or 50 µM. Congo red staining was performed after 6 and 24 h. J774A.1 cells were seeded in Falcon 8-well culture slides for Congo red staining and black 96-well plates for thioflavin T (ThT) staining at a density of 10^4^ cells/cm^2^. One day after seeding, cells were polarized to M0, M1 (LPS/IFN-γ), and M2 (IL-4) subsets as described above. For Congo red staining, SAA1 was added once, 24 h after addition of polarization stimuli, in fresh medium to a final concentration of 50 µM. Congo red staining was performed 72 h after addition of SAA1 (19). Cells were heat-fixed and treated with ice-cold methanol for 10 min. Cells were first counterstained with Mayer’s hematoxylin solution for 60 s and washed five times with fresh tap water for a total of 15 min. Slides were then treated with working solution (80% ethanol, 1% NaCl, and freshly added 0.01% NaOH) for 20 min and stained with 0.2% Congo red in working solution for 20 min. Slides were rinsed two times for 10 s with 100% ethanol. To remove all water drops, slides were dipped three times in Neo-Clear^®^. After drying, slides were sealed with Neo-mount^®^ and analyzed with polarized light using an Eclipse Ti-E fluorescence microscope. For quantitative analysis of amyloid formation by ThT staining, SAA1 was added three times; on day 1 (24 h), day 3 (72 h), and day 5 (120 h) post-polarization. Each time, SAA1 was given in fresh medium containing the respective polarization stimuli to a final concentration of 50 µM. Forty-eight hours after the last addition of SAA1, cells were washed once with PBS and fixed in ice-cold methanol for 10 min at 4°C. Amyloid fibrils were stained with 12.5 µM ThT in PBS at room temperature in the dark and after a final wash with PBS, ThT fluorescence was assessed using a Tecan plate reader Infinite M1000 Pro (excitation: 450 ± 10 nm, emission: 490 ± 10 nm, well scanning mode).

### Statistical Analysis

SigmaPlot software (Systat Software, San Jose, CA) was used for all statistical analyses. Analysis of data was performed using the one-way ANOVA followed by Newman-Keuls or Dunnett’s test for multigroup comparisons, Student’s t-test for 2 groups. *P*-values are indicated as follows: *p < 0.05, **p < 0.01, ***p < 0.001.

## Results

### SAA1 Uptake by Macrophages

A rigorous approach to investigate SAA1-induced changes of gene expression is by using DNA microarrays and primary cells. Accordingly, primary peritoneal macrophages were isolated from NMRI mice and identified by CD11b and F4/80 expression (20). Flow cytometric analysis revealed an average of 67% macrophages in peritoneal cells and purification by adherence led to 91% macrophages (**Supplementary Figure S1A**). Expression of macrophage markers was additionally confirmed on day 2 post-isolation by fluorescence microscopy (**Supplementary Figure S1B**). Primary peritoneal macrophages maintained their adherent phenotype for at least 72 h after isolation and no cell death was detected within this time frame (**Supplementary Figure S1C**). Instead, a continuous accumulation of a luciferase substrate produced by live cells was observed (**Supplementary Figure S1D**).

**Figure 1:**
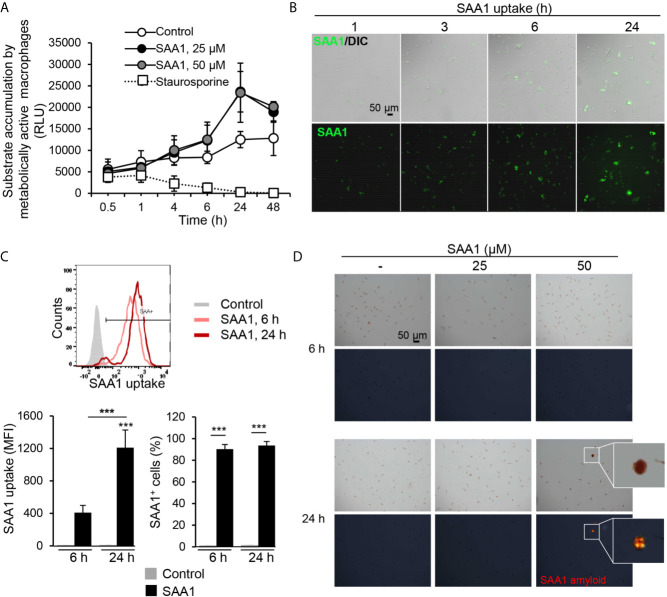
SAA1 uptake by mouse peritoneal macrophages. Peritoneal macrophages were treated with the indicated concentrations of SAA1 for up to 48 h. **(A)** SAA1 treatment does not compromise macrophage viability. Cell metabolic activity was assessed by RealTime-Glo™ MT Cell Viability Assay. Control - vehicle-treated cells. As positive control, cells were treated with 1 µM staurosporine instead of SAA1 (n = 3, mean ± SEM). **(B)** Uptake of SAA1-AF488 (50 µM) as assessed by fluorescence microscopy (magnification 200x). DIC, differential interference contrast microscopy. **(C)** Uptake of SAA1-AF488 (50 µM) as assessed by flow cytometry. The SAA1 uptake graph shows the median amount of SAA1 internalized by a single cell. The right graph shows the number of cells which had internalized SAA1, SAA1-positive cells. Representative images and graphs of the indicated time points are shown (n = 3, mean ± SEM, ***p < 0.001 as analyzed ANOVA followed by Newman-Keuls test). Control - vehicle-treated cells. **(D)** Analysis of SAA1 amyloid formation. Macrophages treated with the indicated concentrations of SAA1 for 6 or 24 h were stained with Congo red and birefringence of amyloid structures was examined using polarized light microscopy. Upper panels - bright light images, lower panels - Congo red birefringence (marked with white square). Representative images are shown. Original magnification 200x.

Murine SAA1 was recombinantly expressed and subsequently purified (for sequence see **Supplementary Figure S2A**). Endotoxin levels in all SAA1 charges were below 0.2 EU/mg protein or 0.01 ng LPS (O55:B5)/mg protein (**Supplementary Figure S2B**), whereas LPS concentrations equal or higher to 0.1 ng/ml, only, activated TNF-α production by murine peritoneal macrophages (**Supplementary Figure S2C**). Thus, at a final concentration of 50 µM (0.58 mg/ml) SAA1 used to stimulate macrophages, endotoxin levels < 0.0058 ng/ml are far below the threshold necessary to activate macrophages. SAA1 did not impair viability of peritoneal macrophages at concentrations up to 50 µM (0.58 mg/ml) (**Supplementary Figure S2D**). SAA1 even increased macrophage metabolic activity at 5 – 50 µM compared to control-treated cells as analyzed by accumulation of luciferase substrate by metabolically active cells (**Figure 1A** and **Supplementary Figure S2D**). Whereas the LPS-induced increase in macrophage metabolic activity was abrogated in the presence of polymyxin B, an antibiotic that binds and neutralizes LPS, the SAA1-induced effects remained unaffected by the addition of polymyxin B (**Supplementary Figure S2D**) clearly indicating that the macrophage stimulation was induced by SAA1.

For DNA microarray analysis, peritoneal macrophages were treated with 50 µM (0.58 mg/ml) SAA1, a concentration commonly reached in plasma during an acute phase response (5). Treatment of peritoneal macrophages with fluorescently-labeled SAA1 led to its rapid cellular uptake, as shown by fluorescence microscopy as early as 1 h after SAA1 addition and also by flow cytometry (**Figures 1B, C** **)**. Already after 6 h of treatment, more than 90% of the cells were SAA1-positive, but after the 24 h, the amount of ingested SAA1 had further increased by approximately 3-times. SAA1 staining with the amyloid-binding dye Congo red revealed first appearance of amyloid structures at 24 h, whereas no amyloid deposits were detected at earlier time points (**Figure 1D** and **Supplementary Figure S3**).

### SAA1 Induces M1-Like Gene Expression in Macrophages

**Figure 2:**
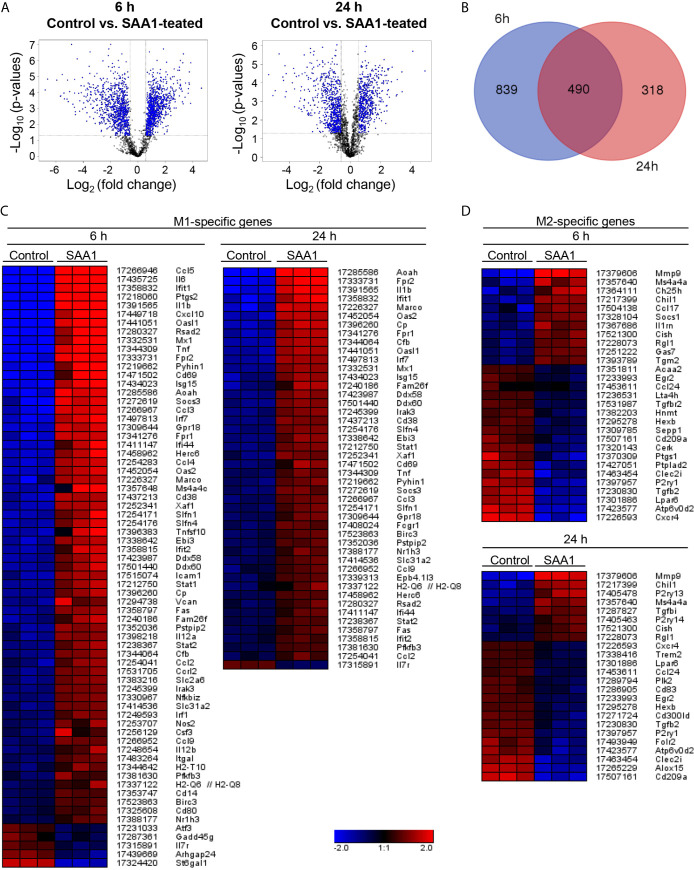
Changes in gene expression induced by SAA1. Mouse peritoneal macrophages were treated with 50 µM SAA1 or vehicle starting on day 2 post-isolation. After 6 and 24 h, cells were harvested and changes in gene expression were analyzed by the Mouse Gene 2.0 ST Array (Affymetrix). **(A)** Volcano plots show the changes in gene expression in peritoneal macrophages after 6 and 24 h of treatment with SAA1. Fold change and corresponding significance of change (*p*-value) between SAA1- and control-treated group are illustrated. **(B)** Venn diagram shows number of genes, the expression of which was changed after 6 and 24 h treatment. Only probe sets with changes ≥ 2 and with a *p*-value < 0.5 have been considered. Data have been obtained from 3 biological replicates. Heatmaps of subset-associated gene expression changes induced by SAA1 treatment in macrophages. Changes in gene expression of M1- **(C)** and M2-associated genes **(D)** are shown as log2(fold change) with the respective probe set ID and gene symbol given on the right. Samples and genes were clustered hierarchically. Mouse peritoneal macrophages were treated with 50 µM SAA1 or vehicle for either 6 or 24 h. Gene expression was analyzed by the mouse gene 2.0 ST array (Affymetrix). Increase (red) or decrease (blue). The three columns per group refer to 3 biological replicates. Control - cells treated with the vehicle for 6 or 24 h, respectively. **(C)** Out of 124 M1-related probe sets, 64 were up- and 5 downregulated after 6 h of treatment. After 24 h, 45 probe sets were up- and 1 downregulated. **(D)** Out of 133 M2-related probe sets, 11 were up- and 18 downregulated after 6 h of treatment. After 24 h, 8 were up- and 16 downregulated.

Based on the time course of SAA1 uptake and amyloid formation, we selected 6 h as an early time point, when the initiated signaling is mediated by soluble SAA1, and 24 h as a later one, when amyloid formation has occurred and changes in gene expression might be influenced by SAA1-derived fibrils. Hence, peritoneal macrophages were treated with SAA1 for 6 and 24 h and gene expression was analyzed on the Affymetrix GeneChip mouse gene 2.0 ST array. Volcano plots for each time point illustrate that numerous genes are differentially influenced by SAA1 (**Figure 2A**). The DNA microarray revealed a total number of 1,647 probe sets that exhibited a change in gene expression of 2-fold and higher and a *p* value ≤ 0.05 (FDR < 0.1). Of those, 1,329 probe sets were changed after 6 h of treatment and 808 at 24 h of treatment with an overlap of 490 probe sets changed at both time points (**Figure 2B**). The raw data can be retrieved at NIH GEO accession viewer using the GEO accession number GSE155278.

**Figure 3:**
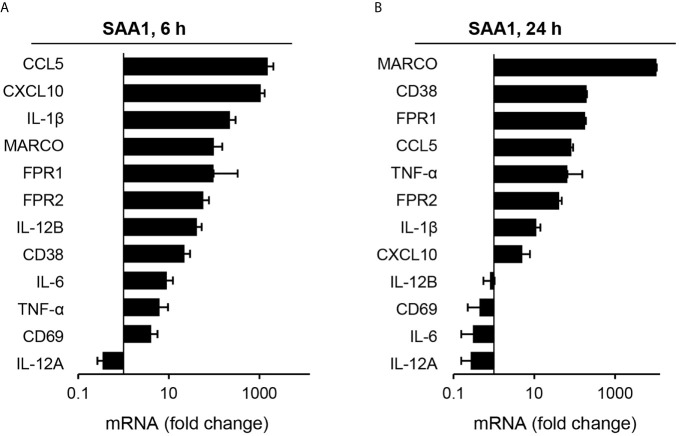
Analysis of gene expression changes induced by SAA1 in mouse peritoneal macrophages by qPCR. Gene expression levels in SAA1-treated macrophages at 6 **(A)** and 24 h **(B)** are given as fold change calculated using the ΔΔ*^CT^* method normalized to *Ubc* and relative to those of control-treated cells. Gene symbols given on the left are sorted by highest upregulation in qPCR at each time point (n = 3, mean ± SEM).

Among all genes regulated by SAA1, genes associated with M1 and M2 macrophage subsets were further investigated. Based on literature data (9, 15, 16, 20–24), a list of M1- and M2-associated genes was created (**Supplementary Tables S1**, **S2**) 'Heatmaps of significant changes in gene expression related to M1- and M2-polarization at 6 and 24 h after SAA1 exposure are shown in **Figures 2C, D**. As shown by the heatmaps in **Figure 2**, SAA1 induces a pronounced upregulation of M1-associated genes at both the early and late time points (**Figure 2A**). **Supplementary Figure S4** shows changes in expression of selected M1-associated genes as quantified by gene array. By contrast, fewer changes were observed for M2-associated genes with a higher number of genes that were downregulated (**Figure 2D**). For a panel of M1-associated genes shown in **Supplementary Figure S4**, amplification of gene transcripts was verified additionally by quantitative PCR. All but one, i.e. IL-12A, of the selected gene transcripts show upregulation either at one or both time points (**Figure 3**). Hence, analysis of M1- and M2-related gene modulation indicates a strong shift towards the M1 subset at early and late time points after SAA1 exposure.

### SAA1 Induces M1-Like Polarization of Macrophages

**Figure 4:**
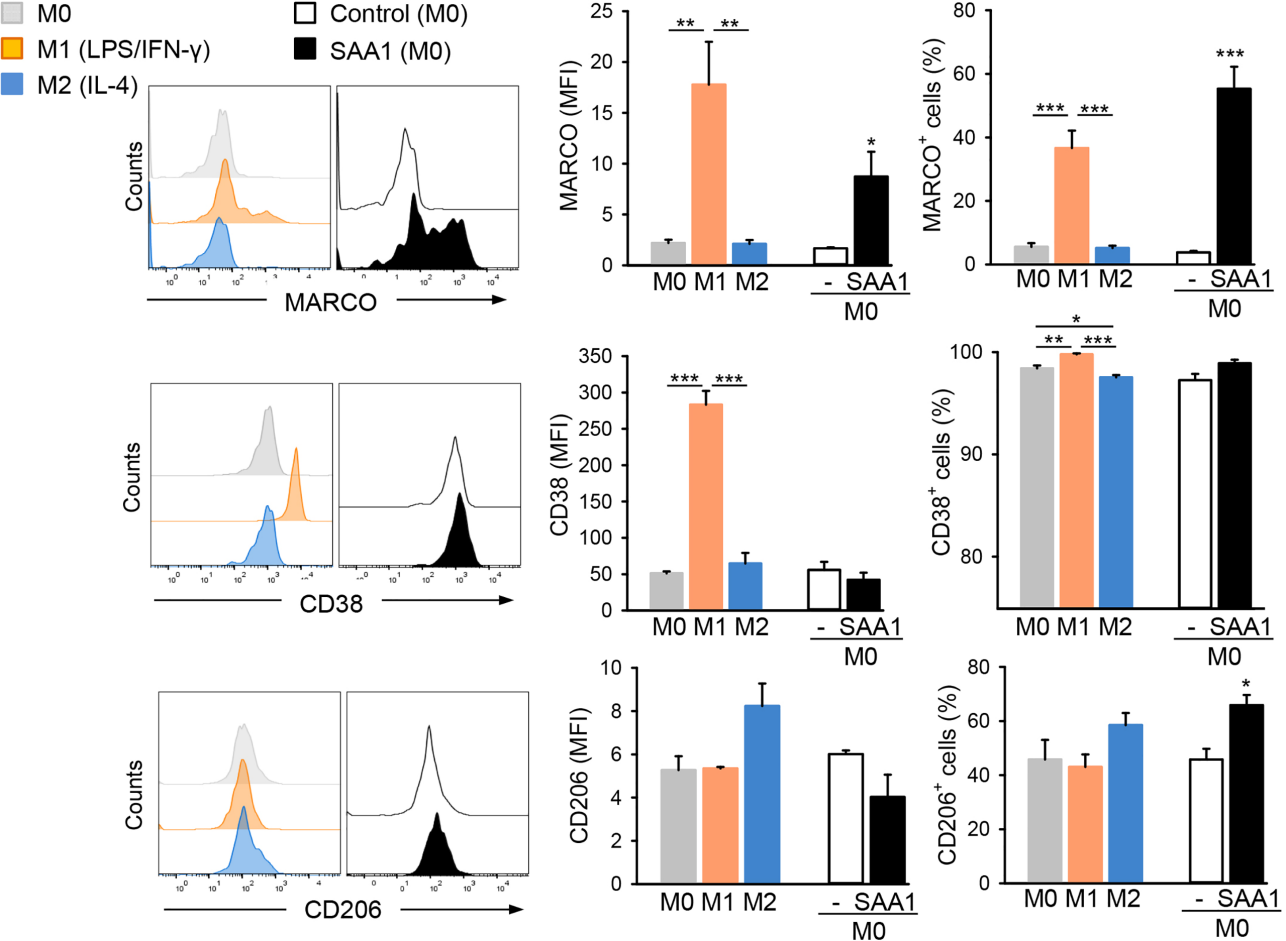
SAA1 increases expression of the M1-related surface marker MARCO by macrophages. Mouse peritoneal macrophages were either left untreated (M0, grey), polarized with LPS O55:B5 (100 ng/ml) and IFN-γ (20 ng/ml) to the M1-like phenotype (M1, orange) or with IL-4 (20 ng/ml) to the M2-like phenotype (M2, blue). Alternatively, non-polarized (M0) macrophages were treated with 50 µM SAA1 (black) or vehicle (-, empty bar) for 24 h. Control - vehicle-treated cells. Expression of the surface markers MARCO, CD38, and CD206 was analyzed by staining with fluorescently-labeled antibodies and subsequent flow cytometric analysis. Left hand graphs show the median expression of a respective protein marker by a cell (MFI, relative median fluorescence intensity). The right hand graphs show the number of cells positive for the above markers (n = 4, mean ± SEM, *p < 0.05, **p < 0.01, ***p < 0.001 as analyzed by one-way ANOVA followed by Newman-Keuls test).

To confirm the above findings at the protein level, expression of macrophage polarization markers affected by the SAA1 treatment was analyzed and compared to that of *in vitro* polarized murine macrophages. Treatment with LPS and IFN-*γ* or IL-4 was used to polarize macrophages to M1-like or M2-like subsets, respectively. Morphological changes, cell surface marker expression, cytokine secretion, as well as production of nitric oxide were analyzed as previously reported for human monocyte-derived macrophage subsets (25). *In vitro* polarization of murine peritoneal macrophages elicited morphological changes within 24 h producing flat and round-shaped M1 and elongated M2 (**Supplementary Figure S5**). Flow cytometric analysis revealed an 8-fold increased expression of MARCO and a 6-fold increase in CD38 by M1 macrophages, as well as an increase in the proportion of MARCO and CD38 positive cells (**Figure 4**). Differently, M2 macrophages exhibited slight, an 1.6-fold, induction of CD206 and no MARCO and CD38 overexpression (**Figure 4**). Treatment with SAA1 triggered a very strong, 5-fold, induction of scavenger receptor MARCO typical for murine M1 macrophages (17) and a 15-fold increase in the proportion of MARCO^+^ cells, but not of CD38. Though, the number of CD206^+^ cells was slightly increased from 46 to 66%, the total number of CD206 receptors was rather decreased after treatment with SAA1 (**Figure 4**). High expression CD206, a mannose receptor, is typical for M2 macrophages (9).

**Figure 5:**
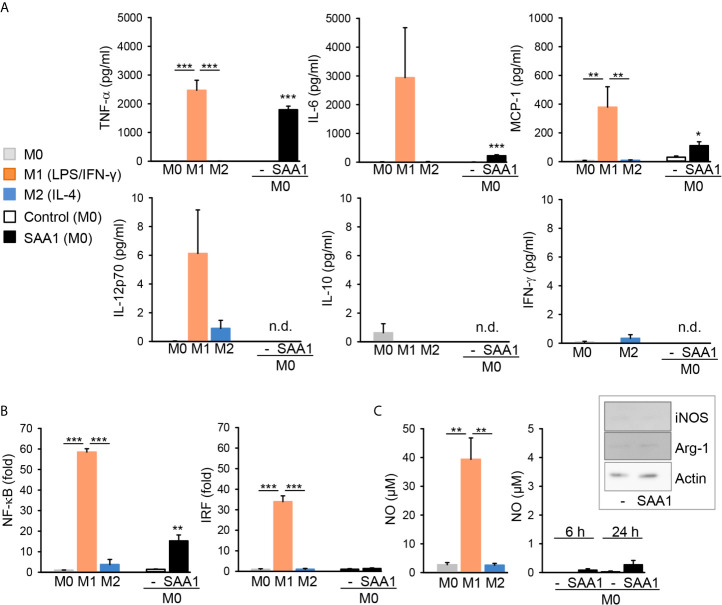
SAA1 induces production of M1-associated cytokines and NF-κB activation by macrophages. Mouse peritoneal macrophages were either left untreated (M0, grey), polarized with LPS O55:B5 (100 ng/ml) and IFN-γ (20 ng/ml) to the M1-like phenotype (M1, orange) or with IL-4 (20 ng/ml) to the M2-like phenotype (M2, blue). Alternatively, non-polarized (M0) macrophages were treated with 50 µM SAA1 (black) or vehicle (-, empty bar) for 24 h. Control - vehicle-treated cells. **(A)** Cytokine secretion was assessed in cell culture supernatants by flow cytometry using cytometric bead assays (n = 4 for control and SAA1, n = 3 for other samples, mean ± SEM, *p < 0.05, **p < 0.01, ***p < 0.001 as analyzed by one-way ANOVA followed by Newman-Keuls test); n.d, below detection limit. **(B)** SAA1 induces NF-κB, but different to LPS/IFN-*γ*-polarized M1 macrophages, it does not induce IRF activation in dual J774A.1 reporter cells (n = 3, mean ± SEM, **p < 0.01, ***p < 0.001 as analyzed by one-way ANOVA followed by Newman-Keuls test). **(C)** At variance to LPS/IFN-*γ*-polarized M1 macrophages (n = 3), SAA1 induces no significant increase in nitric oxide (NO) production as assessed in cell supernatants (n = 4, mean ± SEM, **p < 0.01 as analyzed by one-way ANOVA followed by Newman-Keuls test). Insert: cell lysates were analyzed for NOS2 (iNOS) and arginase-1 (Arg-1) expression by western immunoblotting; actin served as a loading control (n = 2).

Analysis of cytokine release by cytometric bead array revealed that compared to M0 and M2 subsets, classically activated M1 macrophages show characteristic upregulation of TNF-α, IL-6, and MCP-1 release (**Figure 5A**). IFN−*γ*, IL-12 p70, and IL-10 release were below the limit of detection (9.4 pg/ml for IFN−*γ*, 1.9 pg/ml for IL-12 p70 and 3.3 pg/ml for IL-10). The absence of IL-10 in the cell culture supernatant was further verified by ELISA. Low levels of IL-10 are typical for M1 and M2a (IL-4- or IL-13-induced) macrophages, whereas M2b (immune complexes- and TLR agonists-induced) are IL-10^high^ (10).

Confirmation that activation of macrophages by SAA1 is not mediated by an endotoxin contamination, was obtained when macrophages were treated with LPS or SAA1 in the presence of polymyxin B. Polymyxin B completely blocked the LPS-induced TNF-α release, whereas SAA1-induced TNF-α secretion remained unaffected by polymyxin B (**Supplementary Figure S2C**). This confirms the observation that SAA1 was basically LPS-free and that therefore the effects of SAA1 were not mediated by any LPS contamination.

In accordance with the proinflammatory gene induction observed, the proinflammatory master transcription factor NF-κB as well as interferon-regulatory factor (IRF) were strongly activated in M1 (LPS/IFN-γ) macrophages (**Figure 5B**). SAA1 treatment likewise induced NF-κB transcriptional activity, however, to a lesser extent than LPS and IFN−*γ*, which correlated to lower induction of expression of proinflammatory cytokines by SAA1. Differently, no induction of the IRF transcription factor was observed (**Figure 5B**). Additionally, M1 (LPS/IFN-γ) macrophages showed increased production of nitric oxide (NO) compared to M0 and M2 (IL-4) subsets (**Figure 5C**). SAA1-treated macrophages, however, produced no NO. Accordingly, SAA1 did not increase expression of iNOS (NOS2) as well as of ariginase-1 (Arg-1) (**Figure 5C**). High expression of Arg-1 is typical for M2 macrophages (17). The lack of iNOS and NO expression by SAA1-stimulated M1-like macrophages, however, show the difference to LPS/IFN−*γ*-polarized M1 cells.

The murine macrophage cell line J774A.1 is often used in *in vitro* experiments for standardization and avoidance of experimental animals. In addition, robust amyloid formation in J774A.1 can be quantified reliably by ThT fluorescence (3). Yet, the morphology of polarized J774A.1 cells is different from that of primary murine macrophages. In accordance with the literature (26), M1-J774A.1 were elongated and M2-J774A.1 were round-shaped (**Supplementary Figures S5, 6**). In other respects, J774A.1 respond to polarization stimuli and SAA1 similar to peritoneal macrophages.

**Figure 6:**
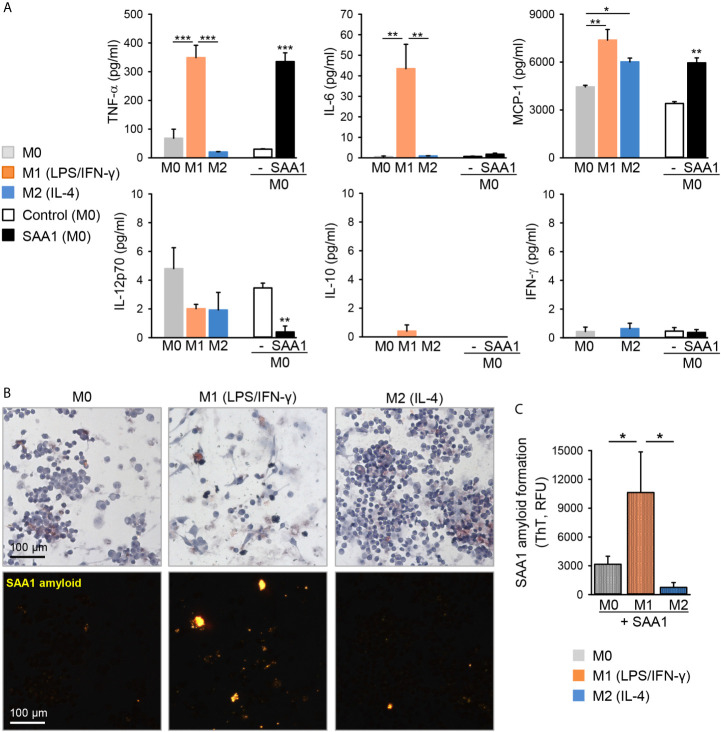
LPS/IFN-γ-polarized M1 macrophages enhance SAA1 amyloid formation. **(A)** J774A.1 macrophages were either left untreated (M0, grey), polarized with LPS O55:B5 (100 ng/ml) and IFN-γ (20 ng/ml) to the M1-like phenotype (M1, orange) or with IL-4 (20 ng/ml) to the M2-like phenotype (M2, blue), or treated with 50 µM SAA1 (black) or vehicle (-, empty) for 24 h. Cytokine secretion was assessed in cell culture supernatants by flow cytometry using cytometric bead assays (n = 3, mean ± SEM, *p < 0.05, **p < 0.01, ***p < 0.001 as analyzed by one-way ANOVA followed by Newman-Keuls test). **(B)** J774A.1 cells polarized for 24 h as described in **(A)** were treated with 50 µM SAA1 for additional 72 h. Samples were stained with Congo red and analyzed by light (upper panel) or polarized light microscopy (lower panel). **(C)** J774A.1 cells polarized for 24 h as described in **(A)** were treated every other day with 50 µM SAA1 for a total of 5 days. On day 7, forty-eight hours after the last addition of SAA1, amyloid fibrils were stained with ThT and analyzed by fluorimetry (n = 3, mean ± SEM, *p < 0.01 as analyzed by one-way ANOVA followed by Newman-Keuls test).

Thus, M0 and M2 macrophages had similar morphological features in both primary as well as J774A.1 cell line-derived macrophage subsets. In analogy to primary macrophages, classically activated M1-J774A.1 cells secreted high amounts of TNF-α, IL-6, and MCP-1 (**Figure 6A**). Likewise, treatment with SAA1 induced the secretion of TNF-α and MCP-1, confirming the M1-polarizing effect of SAA1 in J774A.1 as well (**Figure 6A**). Induction of IL-6 expression by SAA1 was low in primary macrophages and in J774A.1 indicating that LPS and IFN−*γ* are stronger inducers of classically activated macrophages in both cell models.

Overall, macrophages stimulated with SAA1 show a clear M1 macrophage subset-related phenotype confirming our findings on the gene transcript level.

### M1 Polarization Promotes SAA1 Fibril Formation

It is thought that macrophages promote the formation of AA amyloid deposits because they internalize SAA protein, presumably in order to degrade it, but instead of being degraded, the internalized SAA1 misfolds and forms fibril nuclei inside the cells (8). Hence, we have further analyzed if the macrophage polarization state might affect SAA1 amyloid formation. Cells were polarized with LPS/IFN−*γ* or with IL-4 to M1 and M2 subsets, respectively, and thereafter, cells were treated for 3 days with SAA1. Remarkably, neither M0 nor M2 subset promoted formation of SAA1-derived amyloid deposits. Only the M1 macrophages induced deposition of amyloid (**Figure 6B**). The enhanced amyloid forming activity of M1-polarized macrophages was quantified using the amyloid-binding dye thioflavin T (ThT). After treatment for 6 days with SAA1, only the classically activated M1 subset induced amyloid formation, whereas M2 macrophages exhibited the lowest amyloid load compared to M1 and M0 cells (**Figure 6C**). Overall, this indicates that macrophages of the M1 subset promote the formation of amyloid deposits.

## Discussion

In this study we investigated the interplay between macrophages and SAA1 relevant for pathological conditions leading to human morbidity. We show that SAA1 promotes polarization of macrophages towards the classically activated phenotype, whereas, classically activated macrophages in turn promote SAA1 amyloid formation. The results are based on a conclusive DNA microarray approach, which uncovered remarkable upregulation of up to 64 M1-associated genes, whereas the M1-like polarization correlated with the biogenesis of SAA1 fibrils.

As previously shown, SAA1 misfolding starts upon cellular uptake and its targeting to lysosomal compartments for degradation. Within lysosomes, SAA1 is cleaved by proteases such as cathepsins B and L resulting in conformational changes, misfolding, and aggregation (3, 5, 8, 27). Uptake of SAA1 occurred as early as one hour after addition of the soluble protein, whereas extracellular amyloid deposits were first detected after 24 h of incubation. Hence, analysis of gene expression already after 6 h reflects effects induced by soluble SAA1 devoid of endotoxic LPS. Already at this early time point, soluble SAA1 induced macrophage polarization towards the classically activated subset. On the other hand, fibrillary SAA1 might contribute to the predominantly M1-associated gene expression observed after 24 h of treatment with SAA1. Together with an increased NF-κB activity, release of characteristic proinflammatory cytokines, and expression of M1-associated cell-surface receptors, these findings confirm the M1-promoting activity of SAA1. Interestingly, the SAA1-induced shift towards the M1-like macrophage subset was observed at both time points, i.e. with soluble and fibrillary SAA1.

Similarly, others have reported induction of proinflammatory cytokines, such as TNF-α, IL-1ß, and IL-6 by recombinant SAA (12, 28). In addition to secretion of a set of proinflammatory cytokines, they found induction of IL-10 by SAA leading them to the interpretation, that SAA induces M2b-like macrophage polarization (12). Sun *et al.* as well, reported polarization towards the M2 subset by SAA. However, no M1-related gene or marker expression was analyzed in their study (11). We have used three experimental approaches, gene array, cytometric bead array, and ELISA, to prove that SAA1 does not induce any detectable secretion of IL-10 neither in peritoneal macrophages nor in J774A.1 cells. The differences to our study might be explained by the use of human recombinant SAA with murine cells, the use of SAA with an unnatural primary sequence, or neglecting possible endotoxin contaminations as has been analyzed and criticized previously (13).

Additionally, in the above mentioned studies, macrophages had been treated with SAA at nanomolar concentrations. Such low concentrations of SAA are found in plasma of healthy persons (5). In contrast, our study addressed the effect of elevated pathophysiological SAA1 concentrations on macrophages as observed during the acute phase, which is an indispensable prerequisite for SAA1 amyloid formation (1).

Two major signaling pathways regulate synthesis and secretion of M1-associated proinflammatory cytokines, the NF-κB and the NLRP3 inflammasome pathways. It has previously been reported that SAA might activate NF-κB by engagement of TLR-2, TLR-4, or the receptor for advanced glycation endproducts (RAGE) (29–32). However, in all these studies commercial SAA that exerts effects different to that of native SAA (13) had been used. Hence, the respective conclusions have to be interpreted with caution.

As described above, AA amyloidosis can occur as a result of chronic inflammatory diseases with systemically elevated levels of proinflammatory cytokines (5). Hence, in AA amyloidosis, macrophages might already be primed towards the classically activated subset and, as we have shown here, elevated levels of SAA1 could further promote macrophage M1-like polarization. Therefore, we investigated how macrophage polarization might affect amyloid formation. To our knowledge no study has yet analyzed the influence of the macrophage polarization state on the kinetics of amyloid formation. We show here that classically activated macrophages indeed promote SAA1 fibril formation as detected by green birefringence of Congo red stain and fluorescence of ThT bound to fibrillary SAA1.

How classically activated macrophages accelerate formation of SAA1-derived fibrils is not fully clear at this point. In addition to hepatocytes, SAA is known to be expressed by macrophages and some researchers believe that SAA deposits might derive from locally produced rather than from hepatic SAA (33). Furthermore, SAA expression in macrophages can be induced by LPS and proinflammatory cytokines (33). Hence, there might be a feed-forward loop, where SAA stimulates the secretion of proinflammatory cytokines by macrophages, which in turn lead to upregulation of SAA production by macrophages as well as hepatocytes. However, it is mainly the *mSaa3* gene, which is expressed in inflamed tissues (28, 33). It is known from various cell culture models, that the SAA1 concentration is critically linked to the kinetics of amyloid fibril formation (3). However, a role for SAA3 in amyloidosis has not been described yet. At the time points tested, no changes in the *Saa* gene expression were detected by our DNA microarray analyses. Further studies might be required to address this option.

Another explanation, how M1 polarization might favor fibril formation, could be diminished catabolic activity of M1 versus M2 macrophages. Thus, macrophages polarized to M2 with IL-4 exhibit increased phagosomal acidity and cathepsin activity (34). Indeed, uptake of SAA1 and its complete lysosomal catabolism are thought to prevent SAA1 fibril formation (8). Hence, incomplete catabolism of SAA1 by M1 macrophages could be a consequence of lower phagosomal capacity of M1 macrophages accompanied by increased SAA1 load, as it is the case during an acute phase response. Such incomplete SAA1 degradation is believed to generate aggregation-prone intermediates and to promote SAA-derived fibrillogenesis (7, 8, 27).

Our findings might also be of interest for other amyloid diseases. For both, β_2_-microglobulin (β_2_M) amyloidosis and Alzheimer’s disease, representative for systemic and local amyloid diseases, respectively, involvement of macrophages in the pathological mechanisms has been described (35, 36). Whereas macrophages seem to be actively involved in the formation of β_2_M fibrils (35), in Alzheimer’s disease, microglia in the brain might be rather protective by degrading amyloid β (Aβ) plaques (36). However, in both cases fibril-associated macrophages seem to display a rather proinflammatory M1 phenotype (35, 36). Hence, there might be similar pathological mechanisms regarding the role of macrophage polarization in amyloid disease.

In conclusion, the polarizing effect of SAA1 towards a M1-like macrophage subset and the promotion of SAA1 fibril formation by M1 macrophages suggest revision of commonly used cell culture models for AA amyloidosis. We suggest using M1-polarized macrophages as starting point before addition of SAA1 to better mimic the inflammatory *in vivo* situation.

Our results further imply that directing the macrophage phenotype towards the M2 subset, e.g. by biocompatible nanoparticles (25) or the M2 polarizing drug fasudil (37) might be beneficial in the treatment of AA amyloidosis by slowing amyloid formation and disease progression.

## Data Availability Statement

The datasets presented in this study can be found in the following database: NCBI's Gene Expression Omnibus; GEO accession number: GSE155278.

## Ethics Statement

The animal study was reviewed and approved by the Animal Welfare and Ethical Review Board, Ulm University and the respective State Government Agency.

## Author Contributions

Contribution: A-KG, SR, MF, TS, and ThS conceived and designed the experiments. A-KG, SR, SB, and KH conducted experiments and interpreted results. A-KG and TS performed data interpretation and wrote the manuscript; ThS created the study concept, interpreted results, and wrote the manuscript. All authors contributed to the article and approved the submitted version.

## Conflict of Interest

The authors declare that the research was conducted in the absence of any commercial or financial relationships that could be construed as a potential conflict of interest.
