# Supplementary material for: Serum Amyloid A1 Induces Classically Activated Macrophages: A Role for Enhanced Fibril Formation
Source: Front Immunol. 2021 Jun 30;12:691155. doi: 10.3389/fimmu.2021.691155 (PMC8278318; doi:10.3389/fimmu.2021.691155)
Supplement: Supplementary file 1 [file DataSheet_1.pdf]

Supplementary Material

Supplementary Materials and Methods

Quantifications of Endotoxin by EndoLISA

All recombinantly expressed SAA1 batches were tested for contaminations with endotoxin using the EndoLISA® kit (Hyglos, Bernried, Germany) according to manufacturer's instructions. LPS (O55:B5, Sigma) was used for comparison.

Enzyme-linked immunosorbent assay (ELISA)

Peritoneal mouse macrophages or J774A.1 cells were treated with various concentrations of LPS (O55:B5) or SAA1 in combination with polymyxin B (5 µg/ml) for 24 h. Subsequently, cell culture supernatants were tested for TNF-α using the mouse TNF-α DuoSet ELISA (R&D Systems Inc., Minneapolis, MI).

Quantitative PCR

For quantification of mRNA transcripts, RNA samples from peritoneal macrophages treated with or without SAA1 were reverse transcribed using the QuantiTect Reverse Transcription Kit (Qiagen, Hilden, Germany) using the GeneAmp® PCR system 9700 (Applied Biosystem, Foster City, CA) according to manufacturer's instruction. Quantitative real-time PCR of the resulting cDNA was performed using the Luna® universal qPCR Master Mix (NEB, Ipswich, MA). Ubiquitin C (*Ubc*) was used as a housekeeping gene for its stable expression in stimulated mouse macrophages (1). Each quantitative PCR analysis was run in technical triplicates. Real-time PCR primers were purchased from Biomers (Ulm, Germany) and the sequences were as follows:

Ccl5	forward	3'-GTGCTCCAATCTTGCAGTCG-5'
	reverse	3'-CTTCTTCTCTGGGTTGGCACA-5'
Cd38	forward	3'-GAAGACTACGCCCCACTTGT-5'
	reverse	3'-ATGGGCCAGGTGTTTGGATT-5'
Cd69	forward	3'-CACATCTGGAGAGAGGGCAGAA-5'
	reverse	3'-ACAGCCCAAGGGATAGAAACTTG-5'
Cxcl10	forward	3'-ATGACGGGCCAGTGAGAATG-5'
	reverse	3'-TCGTGGCAATGATCTCAACAC-5'
Frp1	forward	3'-GCTGTTGGAAAGTTCAGGAGTC-5'
	reverse	3'-TGAGGTTCACTGCAGACTTGT-5'
Frp2	forward	3'-CTTCATTTACACCACAGGAACCG-5'
	reverse	3'-TTCCATTTTGTCTGCACAACCA-5'
Il12a	forward	3'-CCTTGCATCTGGCGTCTACA-5'

	reverse	3'-TGGAACGCTGACCATAGAGA-5'
Il12b	forward	3'-ACAGAGGAGGGGTGTAACCA-5'
	reverse	3'-AGACATTCCCGCCTTTGCAT-5'
Il1b	forward	3'-GCCACCTTTTGACAGTGATGAG-5'
	reverse	3'-GACAGCCCAGGTCAAAGGTT-5'
Il6	forward	3'-GACAAAGCCAGAGTCCTTCAGA-5'
	reverse	3'-TGTGACTCCAGCTTATCTCTTGG-5'
Marco	forward	3'-CAGGGAAGCAAGGAGCAACT-5'
	reverse	3'-TTGGGGCCAGTGAGACCTAT-5'
Tnfa	forward	3'-GATCGGTCCCCAAAGGGATG-5'
	reverse	3'-CCACTTGGTGGTTTGTGAGTG-5'
Ubc	forward	3'-TCCCACACAAAGCCCCTCAA-5'
	reverse	3'-AGATCTGCATCGTCTCTCTCACG-5'

After initial denaturation (95°C for 60 s) the cDNA was amplified for 45 cycles (each 95°C for 15 s and 55°C for 60 s) on the ABI 7500 Real-Time PCR System (Applied Biosystems, Foster City, CA). Relative mRNA expression is given as $2^{-\Delta\Delta CT}$ normalized to *Ubc* expression using the comparative C_T method (2). Melting curves were generated for each sample to confirm synthesis of a single PCR product.

Microscopy

Bright field images of peritoneal and J774A.1 macrophages were taken in the DIC channel at the indicated time points after polarization using the Eclipse Ti-E fluorescence microscope (Nikon, Tokyo, Japan).

Supplementary Data

Genes associated with M1 and M2 macrophage subsets were created based on literature reports (1-9).

Supplementary Table 1. M1 gene list. Given are the gene symbols and, where available, the respective probe set IDs and gene descriptions as annotated by Affymetrix (GeneChip® Mouse Gene 2.1 ST Array).

Gene Symbol	Probe Set ID	Gene Description
**Adgre5**	-	-
**Ak3**	17363779	adenylate kinase 3
**Aoah**	17285586	acyloxyacyl hydrolase
**Apol6**	17312637	apolipoprotein L 6

Gene Symbol	Probe Set ID	Gene Description
**Apol7a**	17318772	apolipoprotein L 7a
**Apol8**	17318822	apolipoprotein L 8
**Arhgap24**	17439669	Rho GTPase activating protein 24
**Atf3**	17231033	activating transcription factor 3
**Bcl2a1**	-	-
**Birc3**	17523863	baculoviral IAP repeat-containing 3
**Ccl11**	17254053	chemokine (C-C motif) ligand 11
**Ccl15**	-	-
**Ccl19**	17424421	chemokine (C-C motif) ligand 19
**Ccl2**	17254041	chemokine (C-C motif) ligand 2
**Ccl20**	17214857	chemokine (C-C motif) ligand 20
**Ccl3**	17266967	chemokine (C-C motif) ligand 3
**Ccl4**	17254283	chemokine (C-C motif) ligand 4
**Ccl5**	17266946	chemokine (C-C motif) ligand 5
**Ccl8**	17254065	chemokine (C-C motif) ligand 8
**Ccl9**	17266952	chemokine (C-C motif) ligand 9
**Ccr7**	17268972	chemokine (C-C motif) receptor 7
**Ccrl2**	17531705	chemokine (C-C motif) receptor-like 2
**Cd14**	17353747	CD14 antigen
**Cd300lf**	17271776	CD300 antigen like family member F
**Cd38**	17437213	CD38 antigen
**Cd69**	17471502	CD69 antigen
**Cd74**	17350982	CD74 antigen (invariant polypeptide of major histocompatibility complex, class II antigen-associated)
**Cd80**	17325608	CD80 antigen
**Cd86**	17330203	CD86 antigen
**Cfb**	17344064	complement factor B
**Cp**	17396260	ceruloplasmin
**Cpd**	17266157	carboxypeptidase D
**Csf1**	17409075	colony stimulating factor 1 (macrophage)

Gene Symbol	Probe Set ID	Gene Description
**Csf2rb**	17312716	colony stimulating factor 2 receptor, beta, low-affinity (granulocyte-macrophage)
**Csf3**	17256129	colony stimulating factor 3 (granulocyte)
**Cx3cl1**	17504130	chemokine (C-X3-C motif) ligand 1
**Cxcl10**	17449718	chemokine (C-X-C motif) ligand 10
**Cxcl11**	17449725	chemokine (C-X-C motif) ligand 11
**Cxcl15**	17438980	chemokine (C-X-C motif) ligand 15
**Cxcl16**	17265268	chemokine (C-X-C motif) ligand 16
**Cxcl8**	-	-
**Cxcl9**	17449710	chemokine (C-X-C motif) ligand 9
**D14Erd668e**	-	-
**Ddx58**	17423987	DEAD (Asp-Glu-Ala-Asp) box polypeptide 58
**Ddx60**	17501440	DEAD (Asp-Glu-Ala-Asp) box polypeptide 60
**E030037K03Rik**	-	-
**Ebi3**	17338642	Epstein-Barr virus induced gene 3
**Edn1**	17286830	endothelin 1
**Epb4.1l3**	17339313	erythrocyte protein band 4.1-like 3
**F11r**	17219362	F11 receptor
**Fam176b**	-	-
**Fam26f**	17240186	family with sequence similarity 26, member F
**Fas**	17358797	Fas (TNF receptor superfamily member 6)
**Fcgr1**	17408024	Fc receptor, IgG, high affinity I
**Fcgr2b**	17229607	Fc receptor, IgG, low affinity IIb
**Fcgr3**	17229620	Fc receptor, IgG, low affinity III
**Fpr1**	17341276	formyl peptide receptor 1
**Fpr2**	17333731	formyl peptide receptor 2
**Gadd45g**	17287361	growth arrest and DNA-damage-inducible 45 gamma
**Gbp6**	-	-
**Gngt2**	17255466	guanine nucleotide binding protein (G protein), gamma transducing activity polypeptide 2

Gene Symbol	Probe Set ID	Gene Description
**Gpr18**	17309644	G protein-coupled receptor 18
**H2-Q6**	-	-
**H2-T10**	17344642	histocompatibility 2, T region locus 10
**Herc6**	17458962	hect domain and RLD 6
**Hesx1**	17298014	homeobox gene expressed in ES cells
**Hp**	17512809	haptoglobin
**Hsd11b1**	17231229	hydroxysteroid 11-beta dehydrogenase 1
**Icam1**	17515074	intercellular adhesion molecule 1
**Ido**	-	-
**Ido1**	17508188	indoleamine 2,3-dioxygenase 1
**Ifi44**	17411147	interferon-induced protein 44
**Ifit1**	17358832	interferon-induced protein with tetratricopeptide repeats 1
**Ifit2**	17358815	interferon-induced protein with tetratricopeptide repeats 2
**Ifna**	-	-
**Ifnb**	-	-
**Igfbp4**	17256264	insulin-like growth factor binding protein 4
**Il12a**	17398218	interleukin 12a
**Il12B**	17248654	interleukin 12b
**Il15**	17510856	interleukin 15
**Il15ra**	17367004	interleukin 15 receptor, alpha chain
**Il1b**	17391565	interleukin 1 beta
**Il1r1**	17212185	interleukin 1 receptor, type I
**Il23a**	17246091	interleukin 23, alpha subunit p19
**Il2ra**	17366992	interleukin 2 receptor, alpha chain
**IL6**	17435725	interleukin 6
**Il6ra**	17407138	interleukin 6 receptor, alpha
**Il7r**	17315891	interleukin 7 receptor
**Inhba**	17285438	inhibin beta-A
**Irak3**	17245399	interleukin-1 receptor-associated kinase 3

Gene Symbol	Probe Set ID	Gene Description
**Irf1**	17249593	interferon regulatory factor 1
**Irf7**	17497813	interferon regulatory factor 7
**Isf20**	-	-
**Isg15**	17434023	ISG15 ubiquitin-like modifier
**Itga4**	17372307	integrin alpha 4
**Itgal**	17483264	integrin alpha L
**Itgb7**	17322163	integrin beta 7
**Marco**	17226327	macrophage receptor with collagenous structure
**Mos2**	-	-
**Mpa2l**	-	-
**Ms4a4c**	17357648	membrane-spanning 4-domains, subfamily A, member 4C
**Muc1**	17399374	mucin 1, transmembrane
**Mx1**	17332531	myxovirus (influenza virus) resistance 1
**Nampt**	17274875	nicotinamide phosphoribosyltransferase
**Nfkbiz**	17330967	nuclear factor of kappa light polypeptide gene enhancer in B cells inhibitor, zeta
**Nr1h3**	17388177	nuclear receptor subfamily 1, group H, member 3
**Oas2**	17452054	2-5 oligoadenylate synthetase 2
**Oasl1**	17441051	2-5 oligoadenylate synthetase-like 1
**Pdgfa**	17454345	platelet derived growth factor, alpha
**Pfkfb3**	17381630	6-phosphofructo-2-kinase/fructose-2,6-biphosphatase 3
**Pfkp**	17290324	phosphofructokinase, platelet
**Pilr1**	-	-
**Pla1a**	17330359	phospholipase A1 member A
**Psma2**	17285386	proteasome (prosome, macropain) subunit, alpha type 2
**Psmb9**	17343789	proteasome (prosome, macropain) subunit, beta type 9 (large multifunctional peptidase 2)
**Psme2**	17306705	proteasome (prosome, macropain) activator subunit 2 (PA28 beta)
**Pstpip2**	17352036	proline-serine-threonine phosphatase-interacting protein 2

Gene Symbol	Probe Set ID	Gene Description
**Ptgs2**	17218060	prostaglandin-endoperoxide synthase 2
**Ptx3**	17398115	pentraxin related gene
**Pyhin1**	17219662	pyrin and HIN domain family, member 1
**Rsad2**	17280327	radical S-adenosyl methionine domain containing 2
**Saa3**	17491193	serum amyloid A 3
**Sepx1**	-	-
**Slc2a6**	17383216	solute carrier family 2 (facilitated glucose transporter), member 6
**Slc31a2**	17414536	solute carrier family 31, member 2
**Slc7a5**	17513641	solute carrier family 7 (cationic amino acid transporter, y+ system), member 5
**Slco5a1**	17221288	solute carrier organic anion transporter family, member 5A1
**Slfn1**	17254171	schlafen 1
**Slfn4**	17254176	schlafen 4
**Slfn8**	-	-
**Socs3**	17272619	suppressor of cytokine signaling 3
**Sphk1**	17258584	sphingosine kinase 1
**St6gal1**	17324420	beta galactoside alpha 2,6 sialyltransferase 1
**Stat1**	17212750	signal transducer and activator of transcription 1
**Stat2**	17238367	signal transducer and activator of transcription 2
**Tlr2**	17406279	toll-like receptor 2
**Tlr4**	17414836	toll-like receptor 4
**Tnf**	17344309	tumor necrosis factor
**Tnfsf10**	17396383	tumor necrosis factor (ligand) superfamily, member 10
**Tuba4a**	17224540	tubulin, alpha 4A
**Tymp**	-	-
**Vcan**	17294738	versican
**Xaf1**	17252341	XIAP associated factor 1
**Zpb1**	-	-

Supplementary Table 2. M2 gene list. Given are the gene symbols and where available the respective probe set IDs and gene descriptions as annotated by Affymetrix (GeneChip® Mouse Gene 2.1 ST Array).

Gene Symbol	Probe Set ID	Gene Description
**Acaa2**	17351811	acetyl-Coenzyme A acyltransferase 2 (mitochondrial 3-oxoacyl-Coenzyme A thiolase)
**Actn1**	17282226	actinin, alpha 1
**Adk**	17297576	adenosine kinase
**Aldh1a2**	17519112	aldehyde dehydrogenase family 1, subfamily A2
**Alox15**	17265229	arachidonate 15-lipoxygenase
**Amz1**	17444137	archaelysin family metallopeptidase 1
**Anxa4**	17468551	annexin A4
**Aqp9**	17528644	aquaporin 9
**Arg1**	17239845	arginase, liver
**Atp5c1**	17381573	ATP synthase, H+ transporting, mitochondrial F1 complex, gamma polypeptide 1
**Atp6v0a1**	17256473	ATPase, H+ transporting, lysosomal V0 subunit A1
**Atp6v0d2**	17423577	ATPase, H+ transporting, lysosomal V0 subunit D2
**Bcar3**	17402305	breast cancer anti-estrogen resistance 3
**Car2**	17396162	carbonic anhydrase 2
**Cbr1**	17327255	carbonyl reductase 1
**Ccl12**	17254059	chemokine (C-C motif) ligand 12
**Ccl13**	-	-
**Ccl17**	17504138	chemokine (C-C motif) ligand 17
**Ccl18**	-	-
**Ccl22**	17504122	chemokine (C-C motif) ligand 22
**Ccl23**	-	-
**Ccl24**	17453611	chemokine (C-C motif) ligand 24
**Ccna2**	17404821	cyclin A2
**Ccnb1**	17295757	cyclin B1
**Ccnd1**	17498502	cyclin D1

Gene Symbol	Probe Set ID	Gene Description
**Ccne1**	17489886	cyclin E1
**Cd163**	17462843	CD163 antigen
**Cd209a**	17507161	CD209a antigen
**Cd300ld**	17271724	CD300 molecule-like family member d
**Cd83**	17286905	CD83 antigen
**Cdh1**	17505148	cadherin 1
**Cerk**	17320143	ceramide kinase
**Ch25h**	17364111	cholesterol 25-hydroxylase
**Chia1**	17401465	chitinase, acidic 1
**Chil1**	17217399	chitinase-like 1
**Chil3**	17408897	chitinase-like 3
**Chil4**	17408911	chitinase-like 4
**Chn2**	17458692	chimerin 2
**Chst7**	17533535	carbohydrate (N-acetylglucosamino) sulfotransferase 7
**Cish**	17521300	cytokine inducible SH2-containing protein
**Clec10a**	17251978	C-type lectin domain family 10, member A
**Clec2i**	17463454	C-type lectin domain family 2, member i
**Clec4f**	17468209	C-type lectin domain family 4, member f
**Clec7a**	17471541	C-type lectin domain family 7, member a
**Ctsc**	17480018	cathepsin C
**Cxcl13**	17439367	chemokine (C-X-C motif) ligand 13
**Cxcr1**	17224251	chemokine (C-X-C motif) receptor 1
**Cxcr2**	17214142	chemokine (C-X-C motif) receptor 2
**Cxcr4**	17226593	chemokine (C-X-C motif) receptor 4
**Cyfip1**	17478549	cytoplasmic FMR1 interacting protein 1
**Dpysl2**	17307905	dihydropyrimidinase-like 2
**Egr2**	17233993	early growth response 2
**Eif4e**	17403138	eukaryotic translation initiation factor 4E
**Emp2**	17328062	epithelial membrane protein 2

Gene Symbol	Probe Set ID	Gene Description
**Esd**	17301932	esterase D/formylglutathione hydrolase
**Fcer2**	-	-
**Fgl2**	17435089	fibrinogen-like protein 2
**Flrt2**	17277788	fibronectin leucine rich transmembrane protein 2
**Fn1**	17224071	fibronectin 1
**Folr2**	17493949	folate receptor 2 (fetal)
**Gars**	17458771	glycyl-tRNA synthetase
**Gas7**	17251222	growth arrest specific 7
**Gnb4**	17404601	guanine nucleotide binding protein (G protein), beta 4
**Grn**	17256959	granulin
**Hexb**	17295278	hexosaminidase B
**Hnmt**	17382203	histamine N-methyltransferase
**Hrh1**	17461888	histamine receptor H1
**Hs3st1**	17447726	heparan sulfate (glucosamine) 3-O-sulfotransferase 1
**Hs3st2**	17482669	heparan sulfate (glucosamine) 3-O-sulfotransferase 2
**Hspa9**	17353554	heat shock protein 9
**Igf1**	17236288	insulin-like growth factor 1
**Il10**	17216990	interleukin 10
**Il1r2**	17212174	interleukin 1 receptor, type II
**Il1rn**	17367686	interleukin 1 receptor antagonist
**Il6st**	17289889	interleukin 6 signal transducer
**Irf4**	17286295	interferon regulatory factor 4
**Itgam**	17483577	integrin alpha M
**Klf4**	17425401	Kruppel-like factor 4 (gut)
**Krt1**	17322039	keratin 1
**Lipa**	17364114	lysosomal acid lipase A
**Lmna**	17406783	lamin A
**Lpar6**	17301886	lysophosphatidic acid receptor 6
**Lta4h**	17236531	leukotriene A4 hydrolase

Gene Symbol	Probe Set ID	Gene Description
**Maf**	17513289	avian musculoaponeurotic fibrosarcoma (v-maf) AS42 oncogene homolog
**Matk**	17235626	megakaryocyte-associated tyrosine kinase
**Mgl2**	17251959	macrophage galactose N-acetyl-galactosamine specific lectin 2
**Mmp12**	17514495	matrix metallopeptidase 12
**Mmp9**	17379606	matrix metallopeptidase 9
**Mrc1**	17367102	mannose receptor, C type 1
**Ms4a4a**	17357640	membrane-spanning 4-domains, subfamily A, member 4A
**Ms4a6a**	-	-
**Msr1**	17508850	macrophage scavenger receptor 1
**Myc**	17311846	myelocytomatosis oncogene
**Ncl**	17225153	nucleolin
**Ndufa4**	17464836	NADH dehydrogenase (ubiquinone) 1 alpha subcomplex, 4
**Olfm1**	17368646	olfactomedin 1
**Otub1**	17362261	OTU domain, ubiquitin aldehyde binding 1
**P2ry1**	17397957	purinergic receptor P2Y, G-protein coupled 1
**P2ry13**	17405478	purinergic receptor P2Y, G-protein coupled 13
**P2ry14**	17405463	purinergic receptor P2Y, G-protein coupled, 14
**Pdcd1lg2**	17358552	programmed cell death 1 ligand 2
**Pla2g7**	17337796	phospholipase A2, group VII (platelet-activating factor acetylhydrolase, plasma)
**Plk2**	17289794	polo-like kinase 2
**Pmp22**	17250839	peripheral myelin protein 22
**Ppt1**	17418101	palmitoyl-protein thioesterase 1
**Psap**	17233629	prosaposin
**Ptgs1**	17370302	prostaglandin-endoperoxide synthase 1
**Ptpla**	17381892	protein tyrosine phosphatase-like (proline instead of catalytic arginine), member a
**Retnla**	17326069	resistin like alpha
**Rgl1**	17228073	ral guanine nucleotide dissociation stimulator,-like 1

Gene Symbol	Probe Set ID	Gene Description
**Rhoj**	17276386	ras homolog gene family, member J
**Rpl4**	17518298	ribosomal protein L4
**Rps15**	17235292	ribosomal protein S15
**Rps23**	17289031	ribosomal protein S23
**Selenop**	-	-
**Serpinb6**	-	-
**Shmt2**	17245902	serine hydroxymethyltransferase 2 (mitochondrial)
**Sla**	17317637	src-like adaptor
**Slc38a6**	17276273	solute carrier family 38, member 6
**Slc4a7**	17297227	solute carrier family 4, sodium bicarbonate cotransporter, member 7
**Slco2b1**	17480590	solute carrier organic anion transporter family, member 2b1
**Socs1**	17328104	suppressor of cytokine signaling 1
**Socs2**	17244486	suppressor of cytokine signaling 2
**Socs6**	17355873	suppressor of cytokine signaling 6
**Tanc2**	17257405	tetratricopeptide repeat, ankyrin repeat and coiled-coil containing 2
**Tcfec**	-	-
**Tfrc**	17324835	transferrin receptor
**Tgfb1**	17475342	transforming growth factor, beta 1
**Tgfb2**	17230830	transforming growth factor, beta 2
**Tgfbi**	17287827	transforming growth factor, beta induced
**Tgfbr2**	17531987	transforming growth factor, beta receptor II
**Tgm2**	17393789	transglutaminase 2, C polypeptide
**Tiam1**	17331918	T cell lymphoma invasion and metastasis 1
**Tlr5**	17220432	toll-like receptor 5
**Tmem158**	17532485	transmembrane protein 158
**Tpst2**	17440667	protein-tyrosine sulfotransferase 2
**Trem2**	17338416	triggering receptor expressed on myeloid cells 2
**Uba1**	17533604	ubiquitin-like modifier activating enzyme 1

Gene Symbol	Probe Set ID	Gene Description
**Ube2v1**	17394718	ubiquitin-conjugating enzyme E2 variant 1
**Vps35**	17511318	vacuolar protein sorting 35
**Vwf**	17463205	Von Willebrand factor homolog

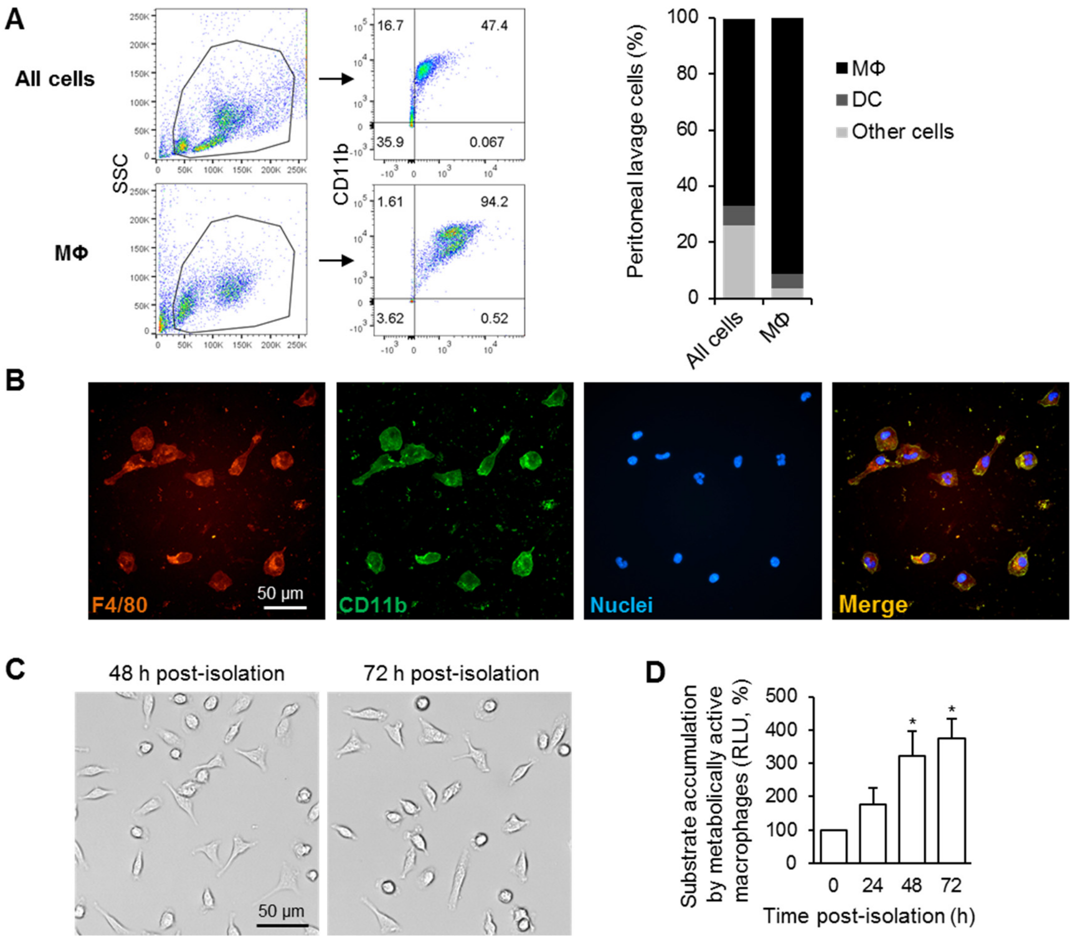

Figure S1│Characterization of mouse peritoneal macrophages. Macrophages were isolated by adherence from peritoneal lavage cells of NMRI mice. **(A)** Phenotype of all peritoneal lavage cells and purified macrophages (MΦ) was assessed by flow cytometry on the day of isolation. Representative plots and graphs are shown (n = 5). CD11b^{+}F4/80^{+} are regarded as macrophages (MΦ), CD11b^{+}F4/80^{-} as dendritic cells (DC), and CD11b^{-}/ F4/80^{-} represent other cell types (others cells). **(B)** Fluorescence microscopy of purified peritoneal macrophages, 48 h post-isolation, stained for CD11b (green) and F4/80 (red). Nuclei were counterstained with DAPI (blue). Original magnification 200x. **(C)** Microscopic analysis of macrophage morphology 48 and 72 h post-isolation. Original magnification 200x. **(D)** Metabolic activity of peritoneal macrophages was assessed by RealTime-Glo™ MT Cell Viability Assay at different time points post-isolation and is shown as mean ± SEM (n = 3, **p* = 0.024 as analyzed by ANOVA followed by Dunnett's test).

A GFFSFIGEAFQGAGDMWRAYTDMKEAGWKDGDKYFHARGNYDAAQRGPGGVWAAEKISDARESFQ
EFFGRGHEDTMADQEANRHGRSGKDPNYYRPPGLPDKY

B

Charge of SAA1	Endotoxin (EU/mg protein)	Equivalent to LPS (O55:B5) (ng/mg protein)
1	0.15	0.0043
2	0.012	0.00024

C

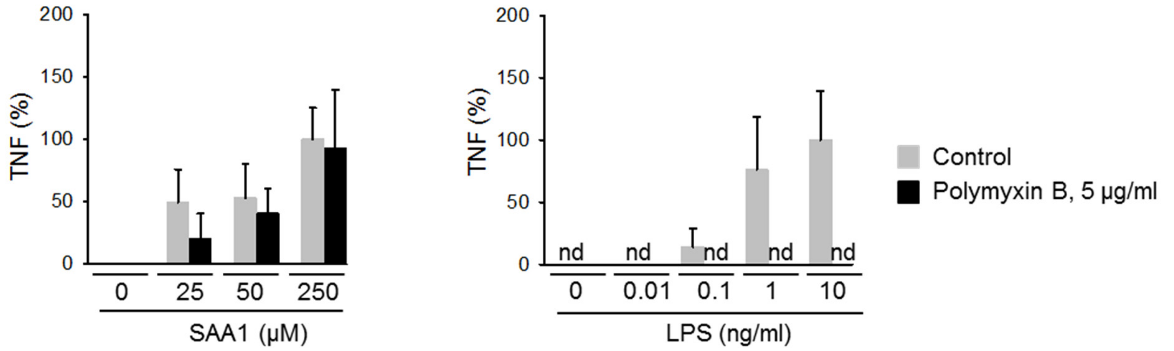

D

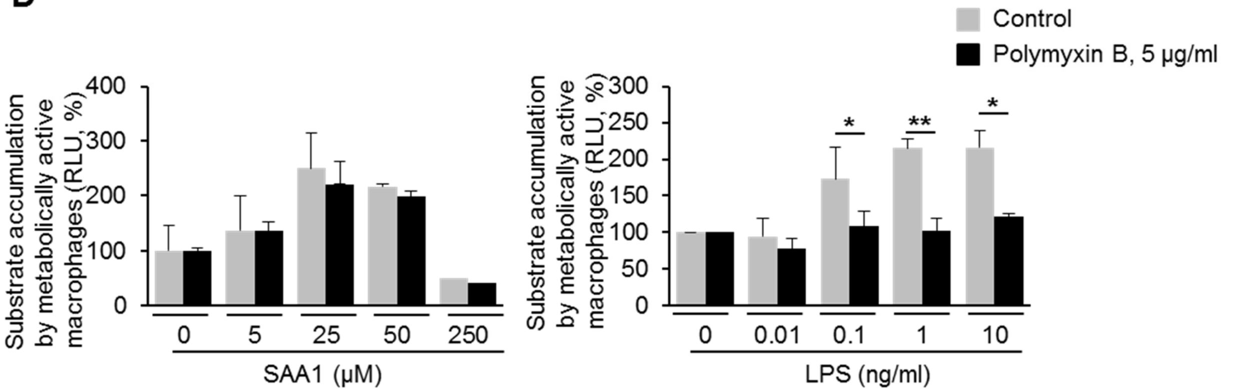

Figure S2│Characterization of recombinant mouse SAA1. **(A)** Sequence of SAA1 (SAA1.1). **(B)** SAA1 contains negligible amounts of endotoxin as analyzed by EndoLISA® (Hyglos). **(C)** LPS at concentrations < 0.1 ng/ml does not activate mouse peritoneal macrophages and stimulation with SAA1 cannot be inhibited by polymyxin B, an antibiotic that binds and neutralizes LPS. Different to LPS, the SAA1 induced TNF-α release and metabolic activity cannot be inhibited by polymyxin B. Peritoneal macrophages were treated with SAA1 (left hand panel) or LPS (O55:B5) (right hand panel) in combination with 5 µg/ml polymyxin B for 24 h. TNF-α secretion was assessed by ELISA. Where indicated, no signal was detected (nd) (LOD = 10 pg/ml); n = 3, mean ± SEM. Control – vehicle-treated cells. **(D)** Effects of SAA1 are not mediated by LPS. Metabolic activity was assessed by conversion of a luciferase substrate using RealTime-Glo™ MT Cell Viability Assay (n = 3, mean ± SEM, *$p < 0.05$, **$p < 0.01$ as analyzed by one-way ANOVA followed by Newman-Keuls test). Control – vehicle-treated cells.

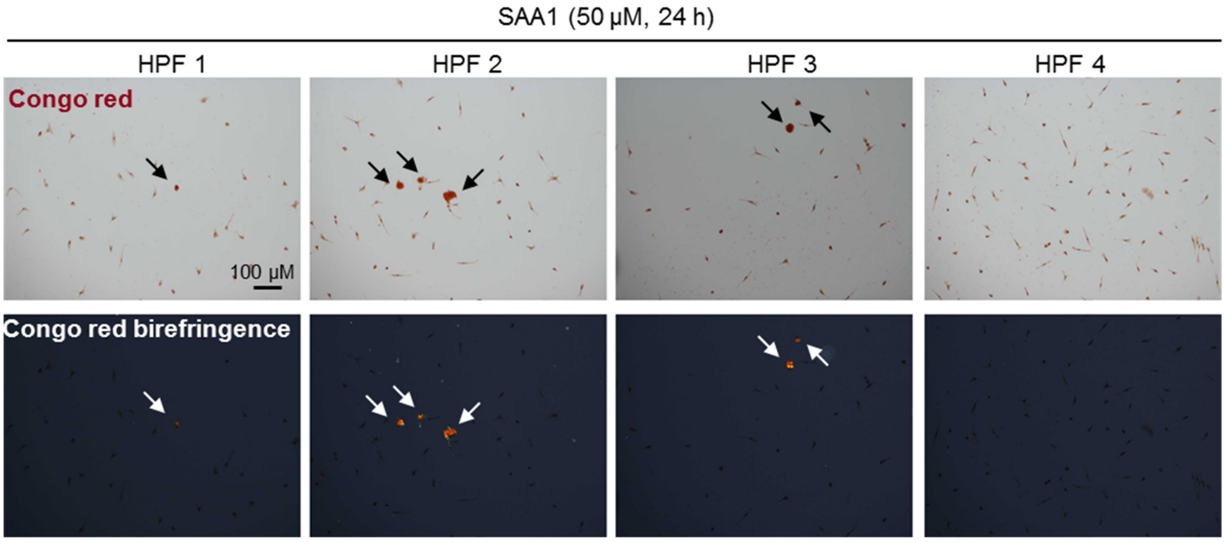

Figure S3 | SAA1-derived amyloid fibril formation by peritoneal macrophages. Additional high power field (HPF) images of peritoneal macrophages treated with 50 µM SAA1 for 24 h. Cells were stained with Congo red and birefringence of amyloid structures was examined using polarized light microscopy. Upper panels show bright light images, lower panels show Congo red birefringence in polarized light.

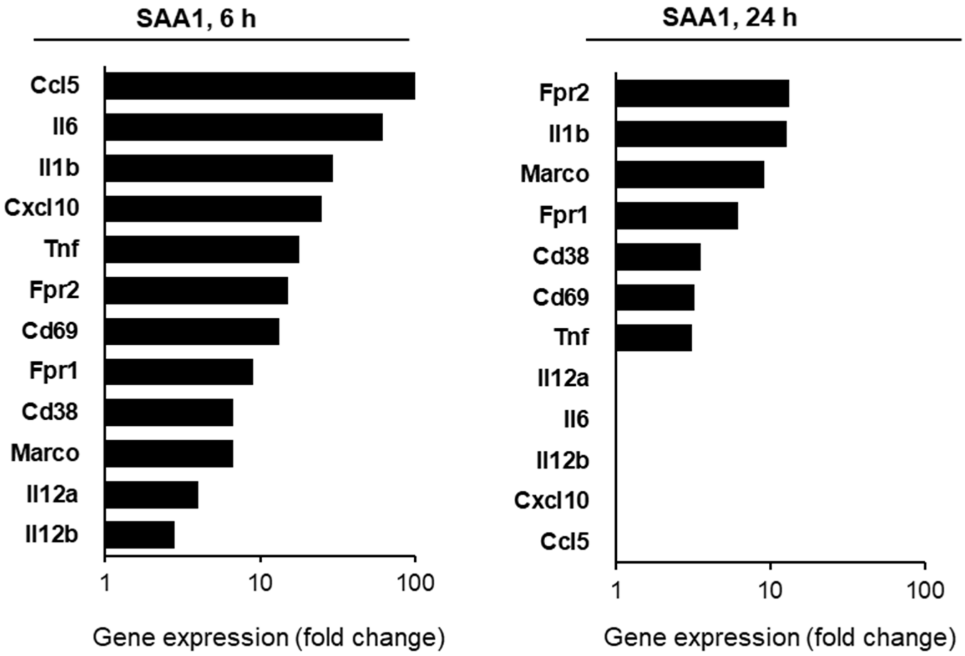

Figure S4 | Changes in selected gene expression induced by SAA1. Mouse peritoneal macrophages were treated with 50 µM SAA1 or vehicle starting on day 2 post-isolation. After 6 and 24 h, cells were harvested and changes in gene expression were analyzed by the Mouse Gene 2.0 ST Array (Affymetrix). Gene expression levels in SAA1-treated macrophages at 6 and 24 h are given as fold change relative to those of control-treated cells. Gene symbols given on the left are sorted by highest upregulation at each time point. Geometric mean values were obtained from 3 biological replicates.

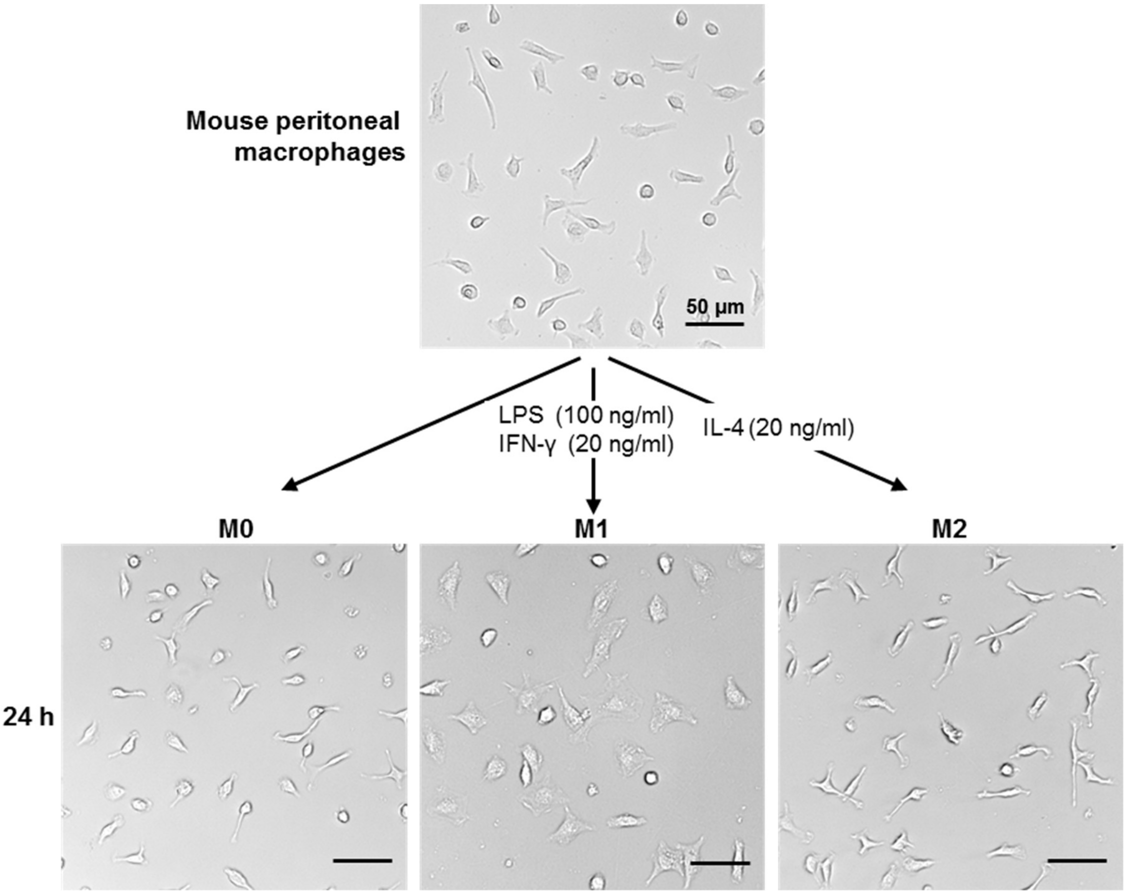

Figure S5 | Scheme of peritoneal macrophage polarization and subset morphology. Murine peritoneal macrophages were polarized towards M1 and M2 subsets or left unpolarized (M0). Microscopic images were taken before and 24 h after polarization (scale bar 50 µm).

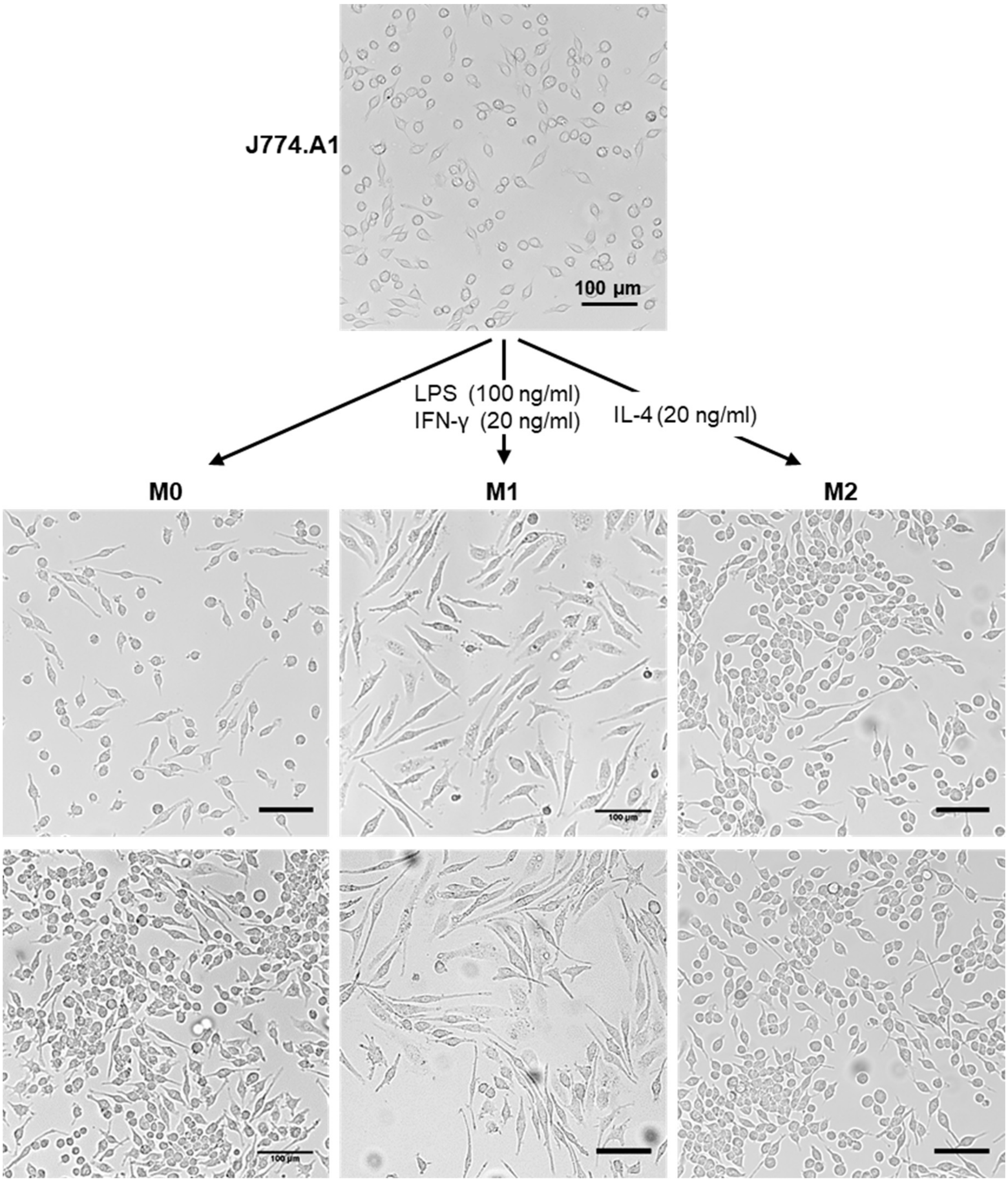

Figure S6│Scheme of J774A.1 macrophage polarization and subset morphology. J774A.1 macrophages were polarized towards M1 and M2 subsets or left unpolarized (M0). Microscopic images were taken before and 24 h or 48 h after polarization (scale bar 100 µm).

References

1. Kalagara R, Gao, W, Glenn, HL, Ziegler, C, Belmont, L, Meldrum, DR. Identification of stable reference genes for lipopolysaccharide-stimulated macrophage gene expression studies. *Biol Methods Protoc* (2016) 1**,** 1-8. doi:10.1093/biomethods/bpw005.

2. Giulietti A, Overbergh, L, Valckx, D, Decallonne, B, Bouillon, R, Mathieu, C. An overview of real-time quantitative PCR: applications to quantify cytokine gene expression. *Methods* (2001) 25**,** 386-401. doi:10.1006/meth.2001.1261.

3. Ghassabeh GH, De Baetselier, P, Brys, L, Noël, W, Van Ginderachter, JA, Meerschaut, S, et al. Identification of a common gene signature for type II cytokine-associated myeloid cells elicited *in vivo* in different pathologic conditions. *Blood* (2006) 108**,** 575-83. doi:10.1182/blood-2005-04-1485.

4. Jablonski KA, Amici, SA, Webb, LM, Ruiz-Rosado, JDD, Popovich, PG, Partida-Sanchez, S, et al. Novel markers to delineate murine M1 and M2 macrophages. *PLoS ONE* (2015) 10**,** 5-11. doi:10.1371/journal.pone.0145342.

5. Mantovani A, Sica, A, Sozzani, S, Allavena, P, Vecchi, A, Locati, M. The chemokine system in diverse forms of macrophage activation and polarization. *Trends Immunol* (2004) 25**,** 677-86. doi:10.1016/j.it.2004.09.015.

6. Martinez FO, Gordon, S, Locati, M, Mantovani, A. Transcriptional profiling of the human monocyte-to-macrophage differentiation and polarization: new molecules and patterns of gene expression. *J Immunol* (2006) 177**,** 7303-11. doi:10.4049/jimmunol.177.10.7303.

7. Martinez FO, Helming, L, Milde, R, Varin, A, Melgert, BN, Draijer, C, et al. Genetic programs expressed in resting and IL-4 alternatively activated mouse and human macrophages: similarities and differences. *Blood* (2013) 121**,** e57-69. doi:10.1182/blood-2012-06-436212.

8. Murray PJ, Wynn, TA. Protective and pathogenic functions of macrophage subsets. *Nat Rev Immunol* (2011) 11**,** 723-37. doi:10.1038/nri3073.

9. Martinez FO, Gordon, S. The M1 and M2 paradigm of macrophage activation: time for reassessment. *F1000Prime Rep* (2014) 6**,** 13. doi:10.12703/P6-13.
